# Hearts in motion: Evaluating the utility of a mobile health unit in Ireland for cardiovascular disease prevention

**DOI:** 10.1016/j.puhip.2026.100835

**Published:** 2026-07-28

**Authors:** N. Briggs, B. Lambe, B. Kehoe, A. McGrath

**Affiliations:** Centre for Health Behaviour Research, Faculty of Health Sciences, South East Technological University, Ireland

**Keywords:** Health promotion, Mobile health, Community setting, Cardiac health education

## Abstract

**Objectives:**

Hypertension is the leading cause of cardiovascular disease and premature death worldwide. This study evaluates the effectiveness of the Irish Heart Foundation's Mobile Health Unit (MHU) in identifying individuals unaware of their elevated blood pressure (BP) and in promoting health awareness through free community-based heart health checks.

**Study design:**

A prospective evaluation study with baseline assessment and six-week follow-up of participants attending the MHU at 14 locations across Ireland.

**Methods:**

Three hundred participants received free BP checks from the MHU. Baseline questionnaires assessed health status, lifestyle behaviours, and health service engagement. Follow-up phone calls were conducted six weeks post-health check (n = 182) to evaluate changes in awareness, behaviour, and GP engagement.

**Results:**

The MHU reached a diverse sample (ages 18-84) with even urban-rural representation. Of participants, 48% were not in current employment, and 56% had not attained further education. Nearly 40% had no prior awareness of their BP levels, 22.1% were diagnosed with high BP and 42.9% were prehypertensive, with significantly more males (34.2%) than females (14.4%) having high BP (p < 0.001). At six-week follow-up, 70% of those referred attended GP visits, resulting in 28.2% undergoing additional tests, 10.3% receiving hypertension diagnoses, and 42.3% making lifestyle changes. Convenience was the most cited reason for participation (34.4%).

**Conclusions:**

The MHU effectively engages at-risk groups through opportunistic health checks, contributing to increased BP awareness, positive behaviour changes, and enhanced health service engagement. Findings support service expansion to underserved areas, with recommendations for increased funding, economic evaluation, and targeted reach strategies.

## 1 Introduction

Cardiovascular disease (CVD) is the leading cause of death across the globe for the past two decades. [1] Despite being a modifiable risk factor, hypertension is the primary cause of CVD and premature death worldwide. [2,3] The latest evidence suggests the prevalence of hypertension has doubled to one billion people globally from 1990 to 2019, exacerbated by a host of complex environmental and behavioural determinants.[3,4] Along with biopsychosocial impacts, hypertension poses a significant economic burden for populations and governments, [5] calculated in the region of several dozen billion USD in direct amounts, [6] a figure which grows exponentially when considering broader societal costs. [7]

In Ireland, recent research from the Irish Longitudinal Study on Ageing (TILDA), suggests the prevalence of hypertension in adults over 50 years was almost 77%, with higher prevalence with advancing age, among males and those with lower educational attainment and physical activity levels. [8] There is limited evidence that disparities in the risk of primary hypertension can be explained solely by genetic factors. [9] Rather, sociodemographic, environmental and behavioural factors are likely to be the main contributors with evidence indicating that hypertension is directly associated with social determinants of health. [10] Lower socioeconomic status has been linked to an increased risk of hypertension based on determinants such as income, occupation, and education. [11,12] Hypertension is therefore complex, and further compounded by its insidious progression where an estimated 46% of adults are unaware that they have high blood pressure (BP). [13] Multi-faceted and targeted approaches are needed to combat the rising prevalence and to reach the global target of reducing hypertension by 33% by 2030. [13]

Hypertension can be detected at the primary health-care level [14] and opportunistic health checks have demonstrated efficacy in the early detection of disease. [15] While these health checks are useful for increasing awareness of hypertension in the general population, the generalisation of such findings should be interpreted with caution as they may not represent disadvantaged or underserved populations. [16] Moreover, those most at risk of hypertension face complex barriers when they seek to engage with health services. [17] Positively, targeted and community focused outreach programmes have demonstrated promise in the ability to engage with more vulnerable populations. [[18], [19], [20]]

Mobile health units (MHU), alongside targeted BP awareness campaigns, represent a particularly effective model for addressing these barriers. This outreach approach provides several benefits for communities and individuals who have limited access to traditional health services by improving access, increasing convenience and removing cost barriers, improving health outcomes through early detection. [21,22] The Irish Heart Foundation's (IHF) MHU exemplifies this model. The unit has been involved in a number of community-based initiatives which sought to engage subpopulations of socially disadvantaged groups such as ‘Farmers have Hearts' in farmers marts and ‘Sheds for Life’ (SFL) in Men's Sheds settings. [23,24] Alongside BP checks and advice, the MHU service aims to raise awareness of the risks for hypertension and to identify high BP in individuals. Participants are offered lifestyle advice by a nurse trained in motivational interviewing. Those who present with a BP reading outside of recommended range are signposted to their general practitioner (GP) for further investigation.

While a previous evaluation of the MHU demonstrated positive outcomes, [20] an updated evaluation is warranted to assess its ongoing relevance, reach and impact within a changing healthcare and population context. Capturing feedback on experiences of MHU engagement and subsequent health behaviours and lifestyle changes provides necessary data to identify if it remains relevant to those it serves. This paper details an evaluation of the IHF's MHU Services with a view to demonstrating the utility of this targeted intervention model in CVD prevention.

## 2 Methods

This study aimed to evaluate the effectiveness of the Irish Heart Foundation's Mobile Health Unit (MHU) in the following areas:

1. Identifying undiagnosed hypertension and prehypertension among adults attending the MHU.
2. Assessing subsequent health-seeking behaviour, including follow-up and medical interventions among those signposted.
3. Exploring changes in health-related knowledge and lifestyle behaviours following the health check.
4. Understanding the reach, accessibility, and acceptability of the MHU service, particularly among underserved or at-risk populations.

These objectives were informed by a set of evaluation indicators developed in collaboration with the Irish Heart Foundation to assess programme impact across detection, engagement, behaviour change, and service satisfaction.

### 2.1 About the mobile health unit

The IHF's MHU travels around the country offering heart health checks to members of the public over 18 years of age. The free health check is 10 minutes in duration and includes BP and pulse checks in a one-on-one consultation with a registered nurse within the unit. The IHF nurse provides heart health information and lifestyle advice, facilitating behaviour change interventions using motivational interviewing techniques and providing a person-led brief intervention with appropriate signposting. All IHF nurses have completed the Irish Health Service Executive's (HSE) Making Every Contact Count (MECC) training and provide behaviour change advice using the MECC framework. [25] The nurse uses an IHF cardiovascular guideline protocol based on the European Society of Cardiology guidelines to assess BP risk. [26] The checks are not diagnostic but seek to identify risk in the community and sign post to a GP or the IHF nurse support helpline.

### 2.2 Study population and sampling

The study population for this evaluation were participants who received a 10-min free nurse-led BP and pulse check as part of the MHU service. Data was collected at baseline from 300 participants across 14 diverse locations including libraries, community centres and Men's Sheds. This sample size was calculated to be representative of the total annual health check population, where the total annual checks (MHU only) in 2023 amounted to 7480. This means 258 surveys were needed to have a confidence level of 95% that the real value is within ±6% of the surveyed value. A researcher approached study participants when they exited the MHU vehicle, explained the study and obtained informed consent to record their BP result and to conduct an interviewer-administered questionnaire on the day and again six weeks later. Ethical approval for the study was granted by South East Technological University Research Ethics Board, Ireland.

### 2.3 Data collection and analysis

Two full-time researchers were trained to conduct face-to-face interviews with participants using structured questionnaires. Following completion of each questionnaire, the next attendee exiting the MHU was approached by a researcher and invited to participate. All data were collected from June to August 2023. Once the participant had completed their BP check and informed consent was obtained, the researcher recorded their BP reading and conducted the interview using the baseline questionnaire. The baseline questionnaire consisted of predominantly closed questions utilising bespoke measures from previous works, [18] divided into the following sections: demographic characteristics, health status and health service engagement, BP knowledge and awareness and lifestyle behaviours. A total of 300 baseline questionnaires were completed. A researcher conducted a follow-up questionnaire with participants six weeks later via telephone. A six-week follow-up period was selected to provide participants with sufficient time to act on advice received during the health check, including attending GP appointments, undergoing further investigations, and initiating lifestyle changes, while minimising recall bias. The timeframe was considered appropriate for assessing the immediate impact of the intervention on awareness, help-seeking behaviour, and early behaviour change.

Of the 300 participants who participated in the baseline questionnaire, n = 182 participated in a follow-up questionnaire at 6 weeks, a follow-up rate of 60.7%. At least two attempts were made to contact each participant before the data collection phase concluded. The follow-up questionnaire was identical to the baseline questionnaire except for the omission of the demographics section and addition of items to measure the outcomes of the BP check. Qualitative open-ended questions were analysed using descriptive content analysis. Quantitative data was analysed using Statistical Packages for the Social Sciences (SPSS). Descriptive statistics were generated to capture characteristics of participants and inferential tests were used to assess differences from baseline to six weeks as well as relationships between variables.

## 3 Results

### 3.1 Socio-demographic characteristics of participants

Of the 300 participants who completed a questionnaire at baseline, the mean age was 59.98 ± 15.15 years, with an age range of 18-87 years. Of these, 44.1% (n = 132) were of retirement age in Ireland (≥66 years). In terms of gender, 40.0% (n = 120) were male while 59.7% (n = 179) were female and one participant (0.3%) identified as ‘other’. The majority of participants (n = 298, 99.3%) identified as ‘white Irish’. Education levels consisted of 12.7% (n = 38) with primary education only, 43.0% (n = 129) with some or completed secondary level education, 33.7% (n = 101) with some or completed third level education and 10.7% (n = 32) with some or completed postgraduate education. A significant proportion (48.0%, n = 144) were not currently in employment, either being retired (29.2%, n = 88) or unemployed (18.8%, n = 56). In terms of marital status, the majority (60.3%, n = 181) were married or cohabiting while 10% (n = 30) reported being ‘in a relationship’ and almost 30% were either separated or divorced (8.5%, n = 24), widowed (10%, n = 30) or single (11.7%, n = 35). There was an almost equal representation of urban dwellers (51.7%, n = 155) versus rural dwellers (48.3%, n = 145).

### 3.2 Health status, access and engagement with health services

The majority of participants (77.3%, n = 232) rated their health as either ‘good’ or ‘very good’. Most participants were non-smokers (85.3%, n = 255), while 14.7% (n = 44) were smokers. Mean fruit and vegetable consumption was 3.35 ± 1.77 daily. Mean weekly consumption of standard alcoholic drinks was 4.0 ± 8.19. The average days spent being physically active for 30 min or more was 4.53 ± 2.44. The majority of participants (55.7%, n = 167) were not meeting the physical activity guidelines at baseline.

Ireland operates a mixed healthcare system where private insurance supplements public provision, while medical cards provide means-tested free access to primary and secondary care. Just under half (49.3%, n = 148) had private health insurance, while 30% (n = 90) had a medical card, 5% (n = 15) had a GP card and 15.7% (n = 47) had no cover. While the majority had visited their GP in the last 12 months (85%, n = 253) 15% either didn't have a GP (0.7%, n = 2) or hadn't been to their GP in over a year (14.7%, n = 44).

In terms of help-seeking, mean scores depicted a positive inclination towards help seeking among the population (see Table 1). Increasing age was associated with delaying help seeking due to cost in time or money (r-0.359, p < 0.001). Males were significantly less likely to seek help for health-related problems when needed compared to females (t = −3.56, p < 0.001).

**Table 1:** Total mean scores for help seeking behaviour among genders at baseline.

Help seeking Statements	All Mean ± SD	Male Mean ± SD	Female Mean ± SD
**I am someone who likes to find out about my health**	8.13 ± 2.64	7.97 ± 2.74	8.26 ± 2.56
**I always seek help for health-related problems when needed**	8.00 ± 2.71	7.35 ± 3.03 **	8.47 ± 2.36
**I sometimes delay seeking help for health problems because I am afraid or uncertain**	2.95 ± 3.44	3.23 ± 3.54	2.71 ± 3.34
**I sometimes delay seeking help for health problems because of the time or money that it costs**	2.24 ± 3.35	2.44 ± 3.49	2.10 ± 3.24
**I am comfortable talking to my doctor about my health concerns**	9.47 ± 1.41	9.44 ± 1.24	9.48 ± 1.52
0 = Strongly disagree, 10 = strongly agree **p < 0.001			

### 3.3 Blood pressure results

The mean systolic BP reading was 127.01 ± 17.25 mmHg, ranging from 84 to 194 mmHg. The mean diastolic reading was 79.17 ± 9.49 mmHg, ranging from 55 to 110 mmHg (see Table 2). Age was positively correlated with BP (r 0.336, p < 0.001). The proportion of people detected to have high BP, as per the IHF protocol, was 22.1% (n = 64; ≥140/90 mmHg), the proportion classed as prehypertensive was 42.9% (n = 124) and the proportion with normal BP was 34.9% (n = 101). There was a significant difference between the proportion of males (34.2%, n = 39) versus females (14.4%, n = 25) who had high BP, even when controlling for age (z = 7.267 p < 0.001).

**Table 2:** Blood pressure results at baseline for the total population and disaggregated by gender.

	All (n = 294)	Males (n = 114)	Females (n = 180)
Mean Systolic BP ± SD (range)	127.01 ± 17.25 (84 to 194 mmHg)	132.64 ± 16.48 (92 to 194 mmHg)	123.38 ± 16.77 (84 to 174 mmHg)
Mean Diastolic BP ± SD (range)	79.17 ± 9.49 (55 to 110 mmHg)	80.31 ± 9.38 (55 to 110 mmHg)	78.43 ± 9.55 (57 to 107 mmHg)
Hypotensive	0.7% (n = 2)	0.0% (n = 0)	1.1% (n = 2)
Normal BP	34.2% (n = 99)	21.1% (n = 24)	42.5% (n = 74)
Pre-Hypertensive	42.9% (n = 124)	44.7% (n = 51)	42.0% (n = 73)
Hypertensive	22.1% (n = 64)	34.2% (n = 39) **	14.4% (n = 25)

**p < 0.001.

A significant proportion (39.5%, n = 118) reported having no prior awareness of their BP levels. Almost a quarter (23.2%, n = 69) were signposted to their GP based on their BP check results and over half (55.9%, n = 38) reported it being their first time to be signposted based on BP. Of those detected as hypertensive, 67.2% (n = 43) were signposted to their GP. Of those in the hypertensive range, 37.5% (n = 24) reported it was their first time being signposted in relation to BP and 31.3% (n = 20) were unaware of their BP status prior to the check. This means that 6.67% (n = 20) of the sample were hypertensive yet unaware.

Participants most commonly cited the convenience of the MHU (34.4%, n = 103) as their reason for getting their BP checked, followed by curiosity (31.1%, n = 31), concerns about BP levels (17.7%, n = 53), and encouragement from family and friends (8.7%, n = 26). Over a quarter (27.1%, n = 81) reported being prescribed medication for BP and taking it as prescribed.

### 3.4 Six-week follow up

Of the 300 baseline participants, n = 182 participated in follow-up at 6 weeks, a follow-up rate of 60.7%. Of these, 30.8% (n = 56) had been advised to visit their GP and 69.6% (n = 39) reported doing so. Of those who visited their GP, 28.2% (n = 11) had further testing, 10.3% (n = 4) received confirmation of hypertension and commenced medication, 7.7% (n = 3) had medication adjusted, and 48.7% (n = 19) had their GP confirm normal BP. One participant (n = 1) reported a referral to an Emergency Department due to a hypertensive crisis which resulted in pacemaker insertion. Of those signposted who did not attend, 41.2% (n = 7) reported being too busy and 41.2% (n = 7) did not think it was necessary.

Following their MHU visit, 42.3% (n = 77) reported making lifestyle changes, including dietary improvements (20.9%, n = 38) and becoming more physically active (19.2%, n = 35). Table 3 highlights positive changes in awareness and knowledge from baseline to follow-up, with significant changes in recognising high BP readings, understanding that physical activity can reduce BP, and knowledge about alcohol and smoking effects on BP (p < 0.01).

**Table 3:** Changes in knowledge and awareness of the aetiology of cardiovascular disease and hypertension from baseline to follow up.

Statements:		Baseline n = 300	Follow up n = 182
*A BP of 120/80 is …*	High	8.3%	3.3%
	Low	4.3%	1.6%
	Normal	62.0%	89.0%
	Don't know	25.7%	6.1%
*A BP of 140/90 is …*	High	64.0%	92.9%**
	Low	2.0%	2.2%
	Normal	8.3%	1.6%
	Don't know	25.7%	3.3%
*Diabetes can cause high BP*	Yes	61.2%	67.6%
	No	12.4%	4.4%
	Don't know	26.4%	28.0%
*High BP can cause a stroke*	Yes	98.0%	98.9%
	No	0.7%	0.0%
	Don't know	1.3%	1.1%
*High BP can lead to a heart attack*	Yes	98.0%	96.2%
	No	0.7%	0.0%
	Don't know	1.3%	3.8%
*Meeting the PA guidelines can lower BP*	Yes	90.0%	97.8%*
	No	3.7%	0.5%
	Don't know	6.3%	1.6%
*Losing weight will make your BP*	Go up	3.3%	0.5%
	Go down	74.7%	83.5%
	Stay the same	8.7%	8.8%
	Don't know	13.4%	7.1%
*Consuming alcohol can make your BP*	Go up	84.7%	98.9%**
	Go down	3.7%	0.0%
	Stay the same	1.7%	0.5%
	Don't know	10.0%	0.5%
*Smoking can make your BP*	Go up	93.3%	100.0%*
	Go down	1.7%	0.0%
	Stay the same	0.3%	0.0%
	Don't know	4.7%	0.0%
*A person who has high BP should consume less salt*	Yes	98.0%	99.5%
	No	0.3%	0.5%
	Don't know	1.7%	0.0%
*A person will always have symptoms of high BP*	Yes	18.3%	8.2%
	No	73.3%	91.8%
	Don't know	8.3%	0.0%

*p < 0.01 **P < 0.001.

Open-ended responses indicated that convenience, accessibility and the opportunity to receive a free health check were the most frequently cited reasons for engaging with the Mobile Health Unit. Participants also highlighted the friendly and supportive approach of staff and valued the opportunity to discuss their health concerns in an informal community setting. Among participants who did not attend a GP following referral, lack of time and perceptions that further follow-up was unnecessary were the most commonly reported reasons.

Overall, there was high acceptability of the MHU approach with mean satisfaction scores of 9.80/10 ± 0.64 for staff approach, 9.56/10 ± 1.05 for location convenience, 9.72/10 ± 0.62 for staff explanations, and 9.47/10 ± 0.82 for written information.

## 4 Discussion

The IHF's MHU Service plays a crucial role in the community by offering free BP checks and health advice to local communities across Ireland. This evaluation examined the reach of the MHU service and its impact on BP detection, health service engagement, health-related knowledge and lifestyle behaviours. The findings demonstrate the positive impact the MHU service has had on participants, achieving its primary aim of identifying undetected hypertension with significant evidence of subsequent health service engagement.

### 4.1 Reach of the MHU service

A core objective of the MHU service is to target underserved and disadvantaged groups. Based on socio-demographic characteristics, this was largely achieved. Almost half of the sample were not in current employment and, even allowing for retirees (29.2%, n = 88), this is considerably higher than recent national unemployment estimates of 4.3%. [27] Additionally, over half of participants had not attained further education. The proportion of participants perceiving their general health as good or very good (77%, n = 232) was slightly lower than the 82-83% reported in recent national surveys, significant considering that lower socio-economic status is a strong determinant of high BP. A notable minority (15.7%, n = 47) had no health coverage, and many had not visited their GP in over a year, suggesting that financial and access barriers may contribute to delayed diagnosis. The MHU did not reach diverse ethnic groups, and highlighting this may prompt a shift in approach. The targeted nature of locations was a deliberate and effective strategy to increase uptake in underserved groups, supported by recent evidence concluding that opportunistic approaches are required to increase uptake among those with higher deprivation levels. [20,23]

This approach also increased uptake amongst men, who are less likely to seek help for health problems than women. This may partly explain why the most common reasons for attending were convenience and curiosity. The proportion of men in the sample (40%, n = 120) is high for health check programmes, [28] further strengthening the rationale for the MHU's opportunistic outreach approach. Similar to previous research, [29] there was a significant difference between males compared to females who were hypertensive. The unit successfully targeted a cohort of men, a group typically less likely to access preventative health services, making this an important achievement in reaching at-risk populations. The significant gender gap in both hypertension prevalence and help-seeking behaviour highlights the value of community-based outreach in reducing access barriers for men. The MHU's ability to attract male participants may serve as a model for engaging men through this targeted settings approach.

### 4.2 Impact of the MHU on blood pressure detection and GP follow up

The MHU consistently delivered positive results in detecting high BP. In this study, 22.1% (n = 64) of follow up participants were found to have high BP, warranting further medical attention, while nearly half were classed as prehypertensive. This high proportion highlights an important opportunity for early intervention through lifestyle modification support. Of the follow up cohort, nearly half of participants reported having no prior awareness of their BP levels and of those in the hypertensive range, one third were unaware of their BP status. This is lower than the estimated 50% of adults with undiagnosed hypertension nationally. [30] This data underlines the important role the MHU plays in detecting undiagnosed hypertension. Mobile units are ideally positioned to offer brief interventions and signposting at this critical early stage of risk.

The standout achievement of the MHU is prompting those with undetected hypertension to engage with health services. Almost a quarter of follow up participants were signposted to their GP for further investigation and over half reported it being their first time to be signposted. During follow-up interviews, the majority of those signposted reported visiting their GP and received interventions including ambulatory monitoring, medication and lifestyle advice, with one emergency referral to hospital resulting in pacemaker insertion. Among those signposted but not attending their GP, the majority of participants reported lack of time and did not think a visit was necessary. Medication compliance remained high, with 100% of participants identified with high BP at follow-up reporting compliance.

### 4.3 Impact of the MHU on knowledge and health behaviour

The educational component of the MHU strives to raise health awareness about risk factors and hypertension management. According to the NCD Risk Factor Collaboration, [31] Ireland has one of the lowest rates of high BP awareness, treatment, and control among high-income countries. Tailored knowledge dissemination approaches such as the MHU's are critical components for successful national hypertension programmes. [32] While participants' health-related knowledge was reasonably good, notable exceptions existed at baseline: approximately one third (n = 114) did not know normal and high BP levels, 39% (n = 116) did not know type 2 diabetes was a risk factor, one quarter did not know weight loss would reduce BP, and 27% (n = 80) thought individuals would always have symptoms. However, marked increases in health-related knowledge occurred at follow-up, suggesting that combining education with opportunistic health checks is effective practice. Recent research recommended lifestyle and behavioural changes as first-line strategies in preventing and treating high BP. [33] The educational component may have encouraged nearly half of participants to make health behaviour changes, with nearly 20% (n = 35) increasing physical activity and over 20% (n = 38) making dietary changes. While numbers were small, two participants quit smoking and two reduced alcohol consumption indicating significant intervention outcomes in relative terms.

### 4.4 Study limitations

Several limitations should be acknowledged. While the baseline sample exceeded the required sample size for the evaluation, only 182 participants completed the six-week follow-up. Although this follow-up rate is comparable to many community-based evaluations, attrition may have introduced response bias, whereby individuals who were more engaged with their health or more likely to have acted on the advice received may have been more likely to participate in follow-up. Consequently, estimates of GP attendance, knowledge improvement, and behaviour change should be interpreted with some caution. Secondly, the short follow-up period may not capture longer-term outcomes. As participants were recruited consecutively rather than through random sampling, response bias and self-reporting bias may have influenced the accuracy of participant-reported information. Self-selection bias is a concern, as those who chose to participate may differ from the wider population. Finally, the findings may have limited generalisability to more ethnically diverse populations.

### 4.5 Conclusion and recommendations

This evaluation demonstrates the substantial effectiveness of MHUs in CVD prevention, particularly for vulnerable and hard-to-reach populations. The MHU's opportunistic outreach approach successfully identified undetected hypertension, with high rates of subsequent GP engagement and appropriate interventions. The educational component improved health-related knowledge and encouraged positive health behaviours, while high participant satisfaction reflected service quality. To maximise impact, continued support for the MHU service is essential, alongside economic evaluation to support scaling up and targeted recruitment strategies to include ethnically diverse populations. Ultimately, MHUs represent an effective, accessible model for hypertension prevention and treatment in underserved populations, advancing community health through early detection and education. Scaling interventions like the MHU across underserved regions could be a critical component in achieving national and global targets for hypertension control and CVD prevention.

## Ethical statement

The study received ethical approval from South East Technological University Research Ethics Board, Ireland.

## Funding

This work was commissioned and funded by the 10.13039/501100001594Irish Heart Foundation. The funders were not involved in the study design, manuscript writing, or collection of data.

## Declaration of competing interest

The authors declare that they have no known competing financial interests or personal relationships that could have appeared to influence the work reported in this paper.
